# Encephaloid Tumour of Antrum—Removal of Superior Maxillary—Recovery

**Published:** 1869-08

**Authors:** Donald Maclean

**Affiliations:** Professor of Surgery and Clinical Surgery in the Louisville Medical College.


### SELECTED ARTICLES.

### ARTICLE V.

### *Encephaloid Tumour of Antrum—Removal of Superior Maxillary—Recovery.*

By DONALD MACLEAN, M.D.,L.R.C.L.,Edinburg.

Professor of Surgery and Clinical Surgery in the Louisville Medical College.

*Case.*—J. W., from Casey county, Ky., æt. 54, married, was introduced to me April 1st, by Dr. Coleman Rogers. Complained of a tumor on the left side of the face, and of which he gave the following brief history: Eighteen months ago, patient noticed that his left nostril was somewhat obstructed; had no pain in the face, but a slight sensation of fullness. Obstruction of the nostril soon became complete, and slight swelling appeared on that side of the nose. General health good. Medical advice was obtained, and various external applications made without effect. Within the last three months, the swelling has increased, especially towards the mouth, and there is now a good deal of deep, gnawing pain all over that side of the face. On examination, the left antrum was found completely filled by a soft, pulpy mass, evidently encephaloid. The anterior wall of the antrum and the hard palate on the left side have both been absorbed, so that the tumor lies immediately beneath the skin of the face and the mucous membrane of the roof of the mouth, adherent to the latter, but fortunately not to the former. The tumor does not appear to involve any of the other bones of the face or cranium. I informed the patient truly as to the progressive and destructive nature of the disease; the advisibility of its immediate extirpation, the excellent prospect of rapid recovery from the operation, and, with no less distinctness, the chances of adverse ulterior contingencies. He at once determined to return, as soon as he had arranged some private matters at home, and submit to the operation; accordingly he again called on me on the 11th of April. In the interval, the tumor was found to have made very considerable progress. The swelling had extended outwards on the face, (i. e., towards the malar bone) and the mouth was considerably fuller. The patient was fully conscious of the progress in these directions, and he complained of more constant and acute pain. Otherwise his health was good. On the 13th, assisted by Drs. Octerlony and Coleman Rogers, and Mr. G. Herchmer, I performed the operation as follows, chloroform having been fully administered by Dr. Octerlony: The first incision extended from the inner angle of the eye, down to the mouth at the middle of the upper lip; from the upper end of this a second was carried outwards along the lower margin of the orbit to the maxillary process of the malar bone. The flap thus formed was reflected, and several enlarged arteries tied as soon as divided, and pressure employed to check the copious hæmorrhage from the general surface of the tumor. The eye and subjacent tissues were carefully dissected from the orbital plate, the mucous membrane of the roof of the mouth divided throughout its whole extent, the bone isolated by sawing and nipping through with the bone pliers; the alveolar, palate, nasal and malar processes, and the pulpy mass, without any difficulty (except what result from its want of cohesion) wrenched out. No vessels were tied in the cavity. The most careful examination convinced all present, viz: besides the gentlemen before mentioned, Drs. Lewis Rogers, Brandeis, Logan. Satterwhite and Turner Anderson, that the whole of the diseased tissue was removed. The cavity was stuffed with lint soaked in a solution of carbolic acid, and the edges of the wound drawn accurately together with silver sutures. The progress of the case after the operation was as satisfactory as could possibly be desired. The wound healed by the first intention. The cavity soon commenced granulating, with every appearance of healthy action. The functions of deglutition and articulation, at first somewhat impaired, were in the course of the first week as fully restored as could be expected under the circumstances. The patient slept and ate well without medicine of any kind; indeed, it may be worth while to mention here that, except a mercurial purge, the day before the operation, one dose of McMunn's elixir immediately after, and one dose of castor oil on the third day after the operation, no medication whatever was resorted to, and this notwithstanding that erysipelas appeared on the face on the fourth day. This complication was just permitted to run its course, as it would have done, even in the face of the most strenuous efforts to arrest it; and at the end of a week it had disappeared, after having passed all over the head and neck, without, however, causing serious inconvenience. On the eleventh day after the operation the patient returned to his home, a distance of more than 100 miles, and on the 31st of April I received a note from him, dated on the 30th, in which he says, "I have improved every day from the time I left you, and am now in fine spirits."

The structure of the tumor, when submitted to microscopic examination, was found to vindicate the opinion which had been confidently expressed, as to its malignant nature.—*Richmond & Louisville Med. Journal.*

